# Potential Role of Mesenchymal Stem Cells in Alleviating Intestinal Ischemia/Reperfusion Impairment

**DOI:** 10.1371/journal.pone.0074468

**Published:** 2013-09-13

**Authors:** Haitao Jiang, Linlin Qu, Rongrong Dou, Lianfang Lu, Sishan Bian, Weiming Zhu

**Affiliations:** 1 Department of General Surgery Ⅱ， Affiliated Hospital of Qingdao University Medical College, Qingdao, China; 2 Department of General Surgery, Shandong Provincial Hospital of Traditional Chinese Medicine, Jinan, China; 3 Research Institute of General Surgery, Jinling Hospital, Nanjing University, Nanjing, China; Rutgers - New Jersey Medical School, United States of America

## Abstract

**Background:**

Transplantation of bone marrow mesenchymal stem cells (MSCs) provides a promising therapeutic efficiency for a variety of disorders caused by ischemia or reperfusion impairment. We have previously demonstrated the efficacy of MSCs in mitigating intestinal ischemia/reperfusion (I/R) injuries in rats, but the mechanism by which MSCs engraft ameliorates I/R injuries has largely been unknown. The present study aimed at investigating probable mechanisms by which MSCs exert their function.

**Methods:**

Male donor derived rat MSCs were implanted into intestine of female recipient rat by direct submucosal injection after superior mesenteric artery clamping and unclamping. The homed MSCs were detected by Y chromosome *in* *situ* hybridization probe, and the tumor necrosis factor-α (TNF-α) content in intestinal mucosa was determined by ELISA. Expression of proliferative cell nuclear antigen (PCNA) in bowel mucosa was assayed by real-time PCR and intestinal mucosa expression of phosphorylation extracellular signal-regulated kinase (pERK1/2) and nuclear factor-κB (NF-κB) were evaluated by western blot.

**Results:**

Four and seven days after MSCs transplantation, the TNF-α content of bowel mucosa in MSCs group was significantly lower than that in saline group. The PCNA in bowel mucosa showed higher expression in MSCs treated group than the saline group, both at 4 and 7 days after cell transplantation. The expression of intestinal mucosal pERK1/2 in MSCs treated group was markedly higher than that in saline group, and the expression of NF-κB in MSCs treated group was noticeably decreased than that in saline group at 4 and 7 days post MSCs transplantation.

**Conclusion:**

The present investigation provides novel evidence that MSCs have the potential to reduce intestinal I/R injuries probably due to their ability to accelerate cell proliferation and decrease the inflammatory response within intestinal mucosa after ischemia and reperfusion.

## Introduction

Since Friedenstein and colleagues first defined mesenchymal stem cells (MSCs) as cells that have the capability of self-renewal and possess multipotency in the 1970s [1], research on these cells has mushroomed for decades. MSCs have been reported not only to be able to differentiate into mesodermal derived lineage, including osteocytes, adipocytes and chondrocytes [2,3,4], but also to have the potential to differentiate into an amazing array of nearly every major cell type in the adult body such as hepatocytes [5,6], pancreatic-like cells [7,8,9] and neuron-like cells [10,11]. Moreover, MSCs are capable of paracrine a variety of cytokines which can apply diverse biological effect. Therefore, MSCs are considered to have the potential for application in a wide variety of degenerative disorders. In addition, MSCs have become attractive candidates in the treatment of many immune disorders because of their low immunogenicity/immune-modulatory properties [12,13,14]. Animal models also demonstrated that MSCs induce the repair of injured organs and ameliorate inflammatory response processes [15,16,17,18]. The encouraging results in such models have initiated the transplantation of MSCs in clinical trials in a range of disorders, including graft versus host disease, inflammatory bowel disease and cases of cardiac infarct [19,20,21].

Intestinal I/R injury is essentially an inflammatory response process and is a significant problem in abdominal aortic aneurysm surgery, small bowel transplantation, cardiopulmonary bypass, strangulated hernias and neonatal necrotizing enterocolitis [22]. Likewise, it is also a common clinical event associated with high morbidity and mortality in both surgical and trauma patients [23]. A number of studies exploring strategies to prevent I/R injuries of the bowel have successfully been applied to attenuate intestinal I/R injury in animal models. Our previous study demonstrated that use of MSCs is a novel modality for reducing intestinal mucosa injury induced by ischemia and reperfusion [24]. That study provided evidence that MSCs infusion has the capability to maintain the morphological integrity of intestinal mucosa, reducing intestinal mucosal permeability, decreasing the incidence of bacteria translocation from bowel lumen to mesenteric lymph nodes, and accelerating the restoration of the gut barrier function after superior mesenteric artery I/R impairment.

A knowledge gap however, still exists regarding the underlying mechanism by which MSCs transplantation reduce intestinal I/R injury. On the basis of our preliminary research and other studies that administered MSCs to I/R injured tissue, we aimed to investigate the influence of MSCs transplantation on regeneration and inflammatory response in intestinal mucosa after I/R injury. We envision that this study will narrow the knowledge gap and lead to a better understanding for the underlying mechanism of MSCs transplantation attenuating intestinal I/R injury, which has mostly been attributed to their potential to inhibit the inflammatory response and to accelerate mucosal cell proliferation.

## Materials and Methods

### Animals

The MSCs donor animals were 4-week-old male Sprague-Dawley (SD) rats. The recipient animals were female Sprague-Dawley rats weighing 180–220 g. All the animals were housed in plastic-bottomed wire-lidded cages and maintained on a 12: 12 hour light/dark cycle in a temperature controlled room (25°C) with free access to water and rat chow. Animals were acclimatized at least 7 days before use. All experimental procedures were carried out in accordance with the *Guide for the Care and Use of Laboratory Animals* published by the National Institutes of Health (NIH publication 86–23, revised 1985), and the protocols were approved by Animal Care and Research Committee of Qingdao University, Shandong Province, China.

### MSCs preparation and surgical procedure

MSCs isolation and expansion was performed according to a previously described method [25]. MSCs culture and identification used the same method as in our previous study [24]. MSCs were cultured using direct adherence and nonadherent cells were removed by changing the culture medium after 72h. Cells were used for transplantation after reaching the third passage of growth. All female recipient SD rats had access only to water for 12 hours prior to the operation. Surgical procedures and cell transplantation were performed as previously described [24,26]. Briefly, 72 animals were randomly divided into 3 groups (MSC, saline and sham groups), each with 24 animals. All the animals were anesthetized with ketamine hydrochloride 100mg/kg introperitoneal injection, midline laparotomy was preformed, and the SMA was dissected and occluded by a microaneurysm clamp. Reperfusion was achieved by removal of the clamp after 45 minutes. In the MSCs group, just after the clamp was released, 1×10^7^ male rat MSCs suspended in 0.5 mL serum free DMEM were submucosally injected into the intestine at 10 different points. In the saline group, animals underwent the same surgical procedure as MSCs group and 0.5 mL of normal saline was submucosally injected into the intestine at 10 different points. However, in the sham group, animals were anesthetized and the SMA was dissected but was not occluded.

### Tracking the donor-derived MSCs in recipient intestine

We used a Y chromosome *in situ* hybridization method (SRY gene detection reagent, Haoyang Biological Manufacture, Tianjin, China) to detect the presence and distribution of MSCs in recipients. Fresh intestinal tissues of the recipient animals were flushed and immediately fixed in 4% paraformaldehyde (4% PFA in 0.1 M PB, phosphate buffer), then dehydrated and the tissue embedded in paraffin wax. 5µm sections were treated with xylene, graded alcohols (95%-80%–60%–30%) and PBS. After incubation with deionized water containing 3% H_2_O_2_ for 10 mins at room temperature, SRY reagent B (concoction fluid) was added for another 10 mins. Sections were then washed with 0.1 mol TBS (PH 7.8) for 5 mins, 0.1 mol TBS for 20 mins at 95~100°C, 0.1 mol cold TBS and 0.2×SSC for 5 mins. The SRY reagent A (hybridization fluid) was used to incubate the sections for 4–8 hrs in humid box at room temperature. After washing with 2×SSC, 0.2×SSC, and 0.1 mol TBS (PH 7.8) at 37°C, the slides were mounted with glycerol and observed at 492nm excitation light under a fluorescence microscope. The positive cells showed green fluorescence.

### Measuring TNF-α content of the intestinal mucosa

Ileal specimen of each experimental animal was removed, intestinal contents were rinsed away with sterile PBS and intestinal mucosal tissues were scraped. After weighing the mucosal tissue and adding homogenization medium in a ratio of 1:10 (weight/volume), intestinal mucosa was fully homogenated at ice water environment. The homogenization liquid was transferred into 1.5ml EP tube, centrifuged at 3000rpm for 15mins, the supernatant prepared for determining the TNF-α using enzyme linked immunosorbent assay reagents (Leino Company, US).

### Real-time PCR for determining proliferating cell nuclear antigen (PCNA) expression in intestinal mucosa

0.1g ileum mucosa of each group was collected in RNase-free tubes and total RNAs of intestinal mucosal tissue was extracted using Trizol reagents (Invitrogen Company, US). cDNA was synthesized using the prime Script^TM^ 1^st^ strand cDNA synthesis kit (Takara, Japan) according to the manufacture’s protocol. In brief, the intestinal mucosa were ground sufficiently using an RNase-free grinding rod in Trizol reagent, then trichloromethane was added to the 1/5 volume of Trizol and mixed thoroughly. The mixture was centrifuged at 14,000 g (4°C) for 15 mins. The supernatants were transferred to new RNase-free tubes and mixed with isopropanol at a ratio of 1:1, then centrifuged at 14,000 g (4°C) for 15 mins after incubation for 10 mins. The supernatants were removed, 1ml 75% ethanol (diluted in DEPC water) was added to the tubes and mixed gently. After centrifugation at 7,500 g (4°C) for 5 mins, the supernatant was removed and the tubes were air-dried at room temperature. To synthesize cDNAs, 5 µl total RNAs and 2×RT buffer 10µl, 1 µl Oligo dT primer (100 µM), 1 µl RT mixture (20 mM) and 13 µl deionized DEPC water was added to each tube. The reaction was under the following conditions: denaturation at 25°C for 10mins, annealing at 40°C for 60 mins, and synthesis at 70°C for 10mins, for 40 cycles. The mixture was then stored at -30°C until use. Real-time quantitative PCR was performed using a sequence detector system (FT2000, Canada) with individual primer pairs and fluorenscently-labbeled probe 50µl reaction according to the reagent (SYBR premix Ex Taq TM II kit Takara, Japan) manufacturer’s protocols. Briefly, real-time PCRs were performed in a total volume of 50 µl, and each reaction contained 2 × PCR buffer 25 µl, 25 µM primers 1.2 µl, 0.4 µl forward and reverse PCR primers, 1 µl cDNA and 22.4 µl deionized DEPC water. The reaction procedure was as following: 94°C for 4 mins, 94°C for 30s, and 60°C for 30s, for 40 cycles. The progress of the PCR amplification was monitored in real-time by fluorescent measurement during each cycle, and the relative concentration of target gene were quantified with its own GAPDH. The analysis of PCR results and calculations was performed using the Rotor-gene software (ver. 6) and the control levels were set at 1.

The sequence of PCNA mRNAs is 5’- GCAACTTGGAATCCCAGAACA, 3’ -CTCCCCACTCGCAGAAAACT, designed by Primer-Primier 5.0 according to the rat PCNA mRNA sequence in GenBank. And the probe sequence was fam+ACAGCTGCGTAGTAAAGATGCCATCTG +tamra

### Western blot to analyze the protein expression of pERK1/2 and NF-kB

Samples (20µg) from intestinal mucosa of each group were electrophoretically separated on 10% SDS polyacrylamide gels and the proteins were then transferred to polyvinylidene difluoride membranes (Amersham Biosciences, RPR303D). The membranes were blocked in blocking solution (0.1% Triton-X-100, 15 mmol/L NaCl, 2 mmol/L Tris-HCl, pH 7.5) containing 3% bovine serum albumin (BSA) for 30mins, then incubated overnight with rabbit anti-pERK1/2 monoclonal antibody (Cell Signaling, US, 1:100), rabbit anti-lamin B1 polyclonal antibody (Abcam, UK; 1:500), and rabbit anti-NF-kB p65 antibody (Santa Cruz, US; 1:100) diluted in Tris-buffered saline with 0.1% Tween 20 (TBST) containing 1% BSA at 4°C, then washed three times (5 mins each time) with TBST. The membranes were incubated with a horseradish peroxidase-conjugated secondary antibody of goat anti rabbit IG (Abcam, UK, 1:1000) for 1 hour, and subjected to a chemiluminescence detection system. Semiquantitative evaluation of bands was assessed densitometrically using the software Quantity One (Bio-Rad).

### Statistical analysis

Data were analyzed using standard statistical software (SPSS 13.0; SPSS Chicago, IL). The values were presented as Mean ± S.D, the statistical significance among groups was evaluated by one-way analysis of variance (ANOVA) followed by post hoc Bonferroni’s multiple comparison test. The significance level was set to *P* < 0.05.

## Results

### Detection of the donor derived MSCs

**Figure 1:**
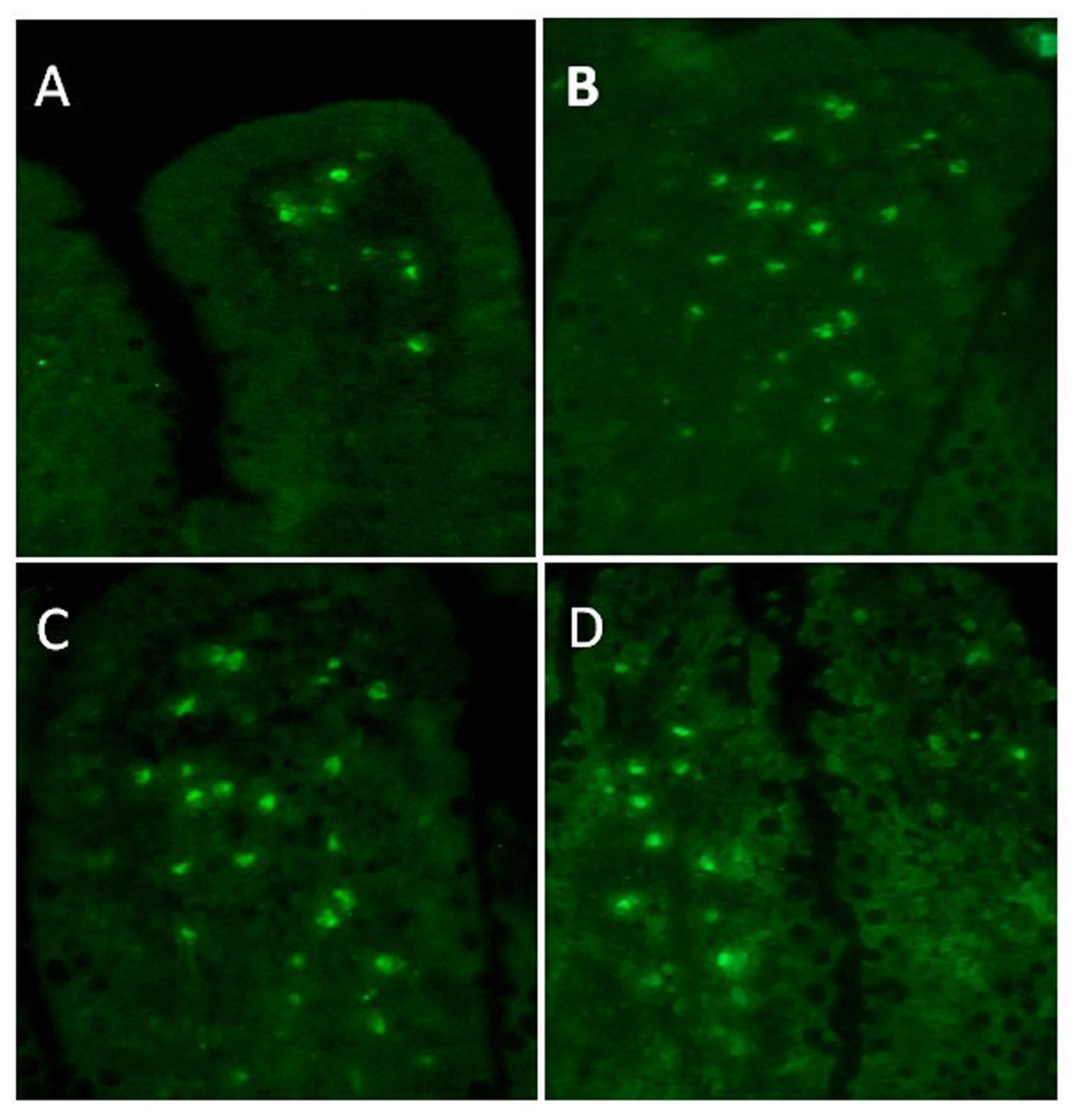
MSCs homing photograph (40×). After cell transplantation, engrafted cells were detected by Y chromosome *in* *situ* hybridization at 1, 4, 7, and 10 days (the green fluorenscent point represents positive donor derived cells), which corresponded to graphs A, B, C, and D respectively. Most of the homed cells were located at mucosal lamina propria, and the amounts of engrafted cells were more at 4 and 7days than 1 and 10 days postoperatively.

After MSCs transplantation, we determined the distribution of donor derived cells in the recipient rat using a Y chromosome *in situ* hybridization method as previously described [24] and selected four time points for identifying the Y chromosome positive cells post transplantation. We found that there were only a few donor derived cells migrating to the lamina propria of intestinal mucosa in recipient rats at 1 day postoperatively. But as time elapsed, more donor derived MSCs engrafted into the intestinal mucosa, with most of the homed cells located at the lamina propria of intestinal mucosa at 4 and 7 days postoperatively. This number of homed cells decreased to a lower level at 10 days postoperatively (Figure **1**).

### TNF-α content in the intestinal mucosa

**Figure 2:**
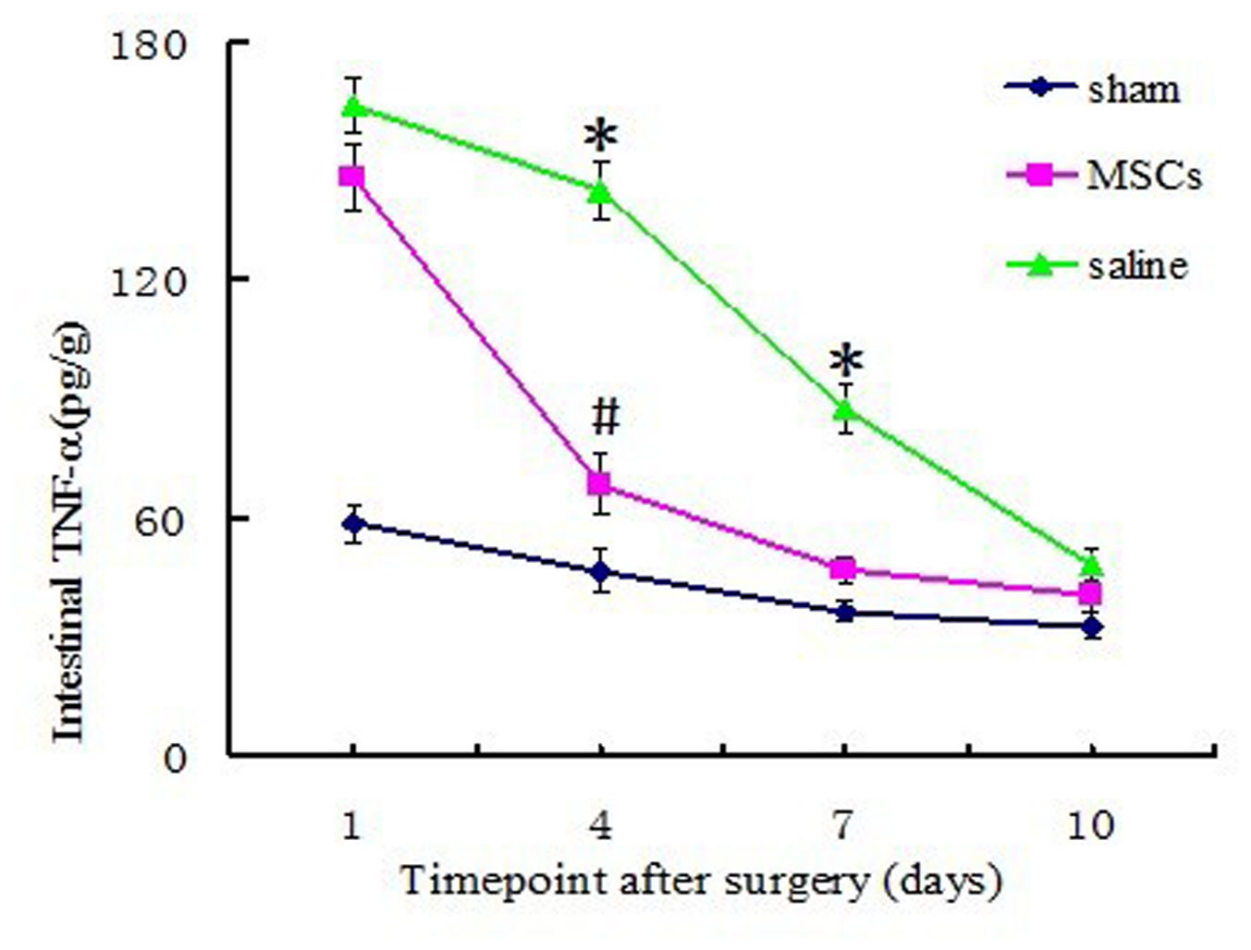
ELISA analysis of intestinal mucosal TNF-α content among the three groups. Data are shown as means ± SD of the three groups, *P < 0.01 vs. MSC group; # *P <0.05* vs. Sham group, values are evaluated by one-way ANOVA test.

TNF-α was an important proinflammatory cytokine during intestinal I/R injuries, and its content in intestinal mucosa of every group was varied depending on the different processing factors. In sham group (rats only receving laparotomy and SMA dissection) mucosal TNF-α was in a low and steady level at each time point postoperatively. However, in both MSCs and saline treated groups, mucosal TNF-α rised to a high level compared to the sham group at 1 day postoperatively, and mucosal TNF-α in saline group went up to an even higher level than that in the MSCs group. 4 days after I/R, mucosal TNF-α in MSCs group and saline group began to decline, and the mucosal TNF-α in MSCs group decreased to a much lower level than that in saline group (P<0.01). This trend was maintained until 7 days postoperatively, at which timepoint, mucosal TNF-α level in MSCs group dropped to the level near that of sham group (P>0.05). However, TNF-α in saline group ramained at a high level until 10 days postoperatively when it finally declined to a level close to that of MSCs group (Figure **2**). From Figure 1 and Figure 2, we can see that the variation of mucosal TNF-α in MSCs group was negatively correlated to the amount of the homed MSCs within the intestinal mucosa, particularly at 4 and 7 days postoperatively: the amount of engrafted MSCs reached a higher level, while the mucosal TNF-α dropped to a lower level.

### PCNA expression in intestinal mucosa of different groups

**Figure 3:**
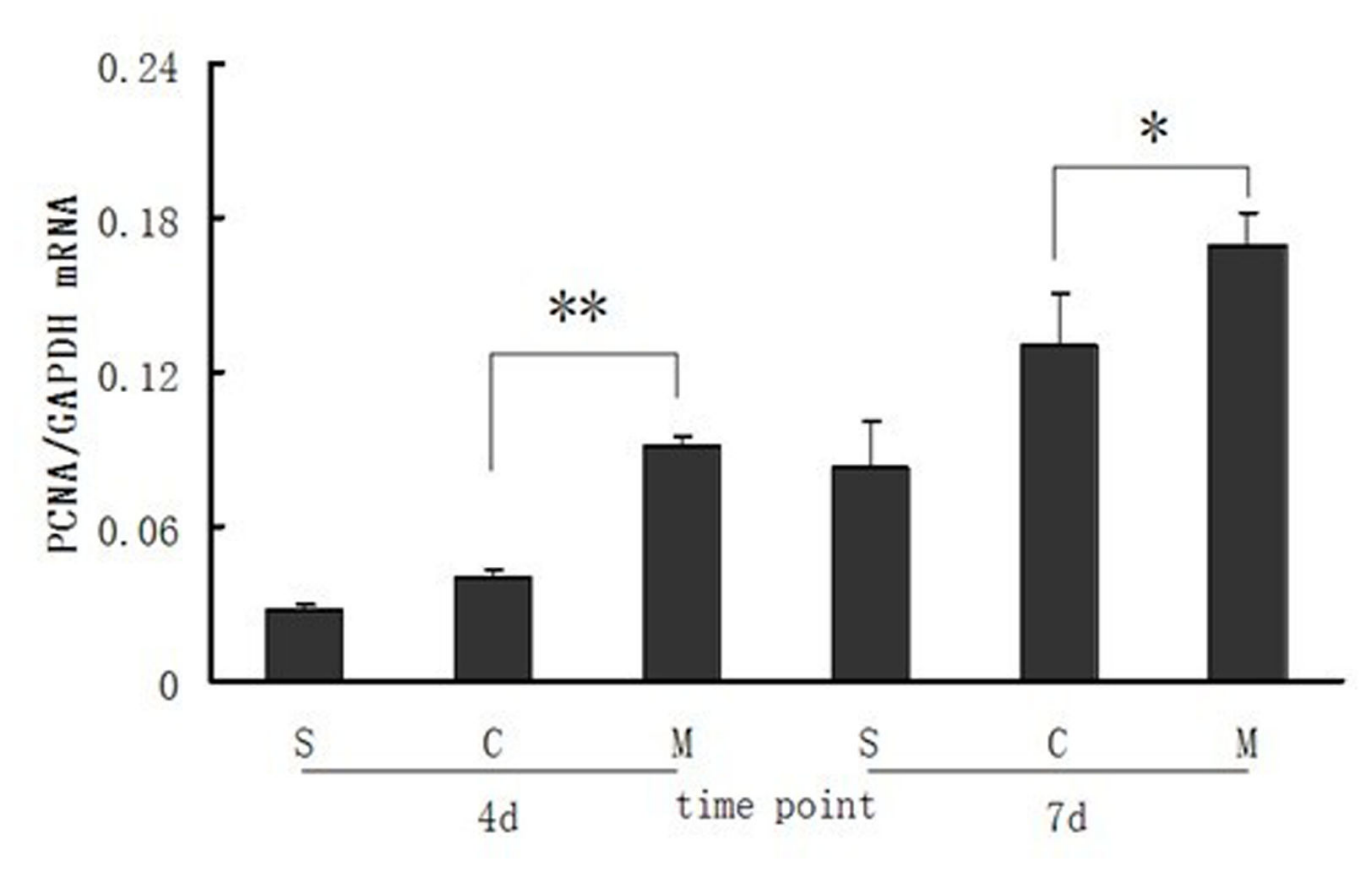
Real-Time PCR analysis of PCNA expression in the intestinal mucosa. Data are means ± SD of each group at different time point and expressed as the ratio of original value mRNA of PCNA to its internal control mRNA of GAPDH. S: sham group; C: control (saline) group; M: MSCs group. 4d and 7d represent 4 days and 7 days postoperatively. ** *P<0.01*, * P<0.05, compared with control (saline) group, by one-way ANOVA test.

Real-time PCR was applied to determine PCNA expression at mRNA level in intestinal mucosa of every group at 4 and 7days after intestinal I/R injuries, which overall showed an increasing tendency of PCNA expression in intestinal mucosa. PCNA in sham group showed a low expression at 4 days postoperatively, and expression of PCNA in saline and MSCs groups were higher than that in the sham group. It is important to note that PCNA expression level in MSCs group was significantly higher than that in the saline group (P<0.01). At 7 days postoperatively, there was still significant difference of mucosal PCNA expression in MSCs group than that in the saline group (P<0.05) (Figure **3**).

### Protein expression of pERK1/2 to tERK1/2 and NF-kBp65 in intestinal mucosa

**Figure 4:**
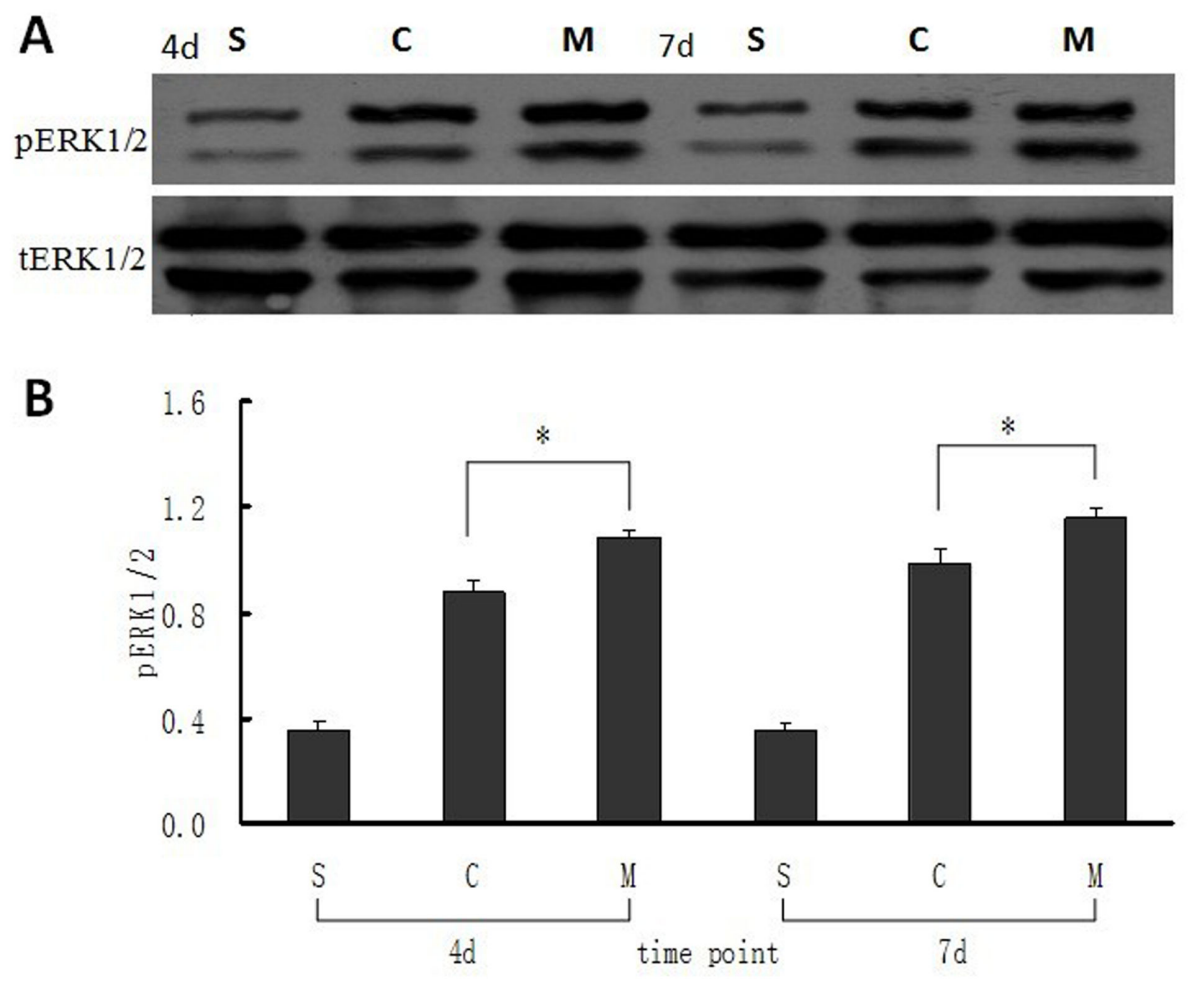
Expression of pERK1/2 in intestinal mucosa at 4 and 7 days after MSCs transplantation. (**A**) Bands of the expression of pERK1/2 and its own tERk1/2 protein were detected by western blot. (**B**) The ratio value of pERK1/2 to its own tERK1/2 of the bands which was evaluated densitometrically using the software Quantity One for the three groups at different time points. Each bar represents mean ± SD of the ratio value in every group. S: sham group; C: control (saline) group; M: MSCs group. 4d and 7d represent 4 days and 7 days postoperatively. *: P<0.05, compared with control (saline) group, by one-way ANOVA test.

**Figure 5:**
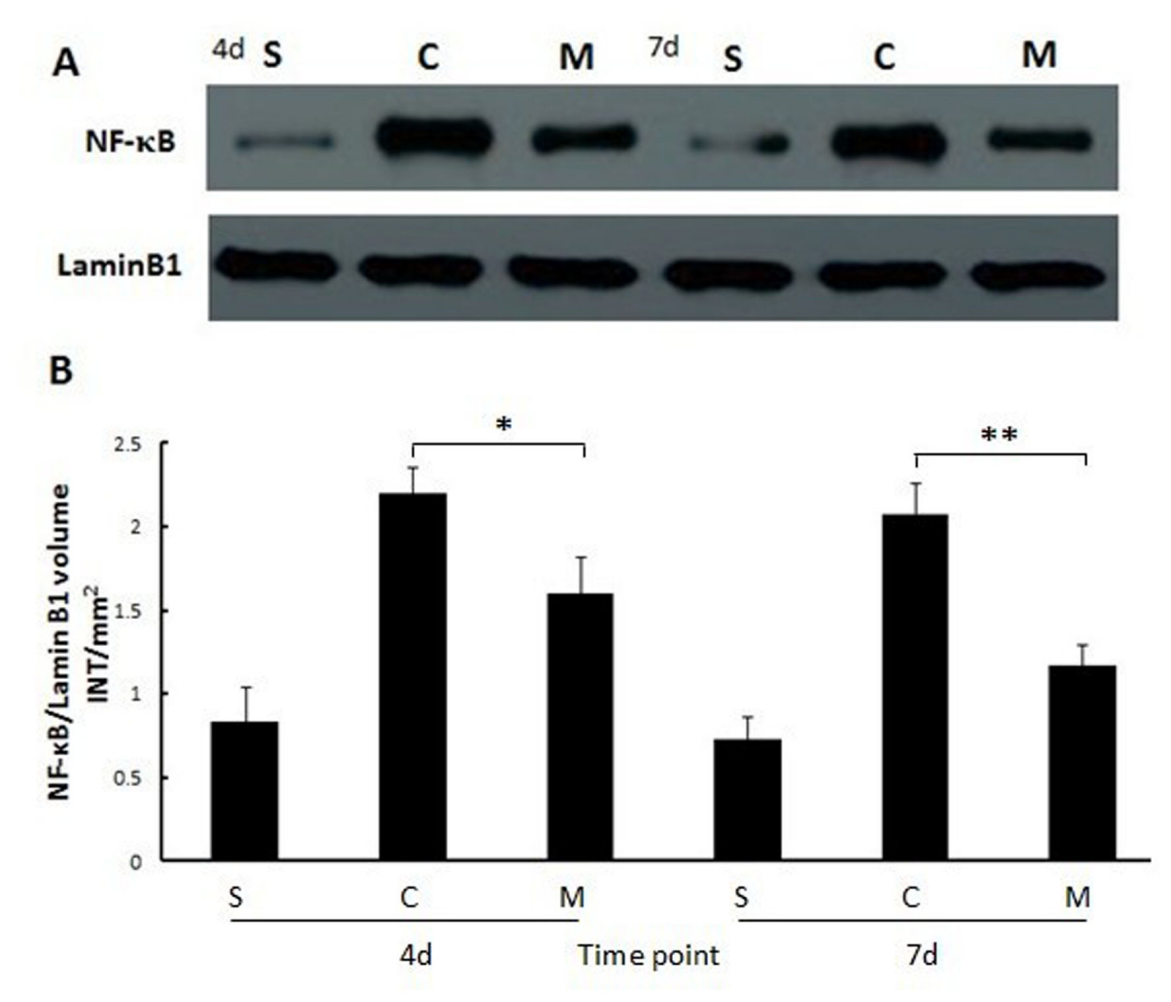
Mucosal NF-κB expression in each group at 4 and 7 days after MSCs administered. (**A**) Semiquantitative assessment of bands for NF-κB and its internal control protein. (**B**) Values of the NF-κB band performed densitometrically using the software Quantity One. Each bar represents the mean ± SD of the different groups; values were compared by one-way ANOVA test, **: P<0.01, *: P<0.05. S: sham group; C: control (saline) group; M: MSCs group. 4d and 7d represent 4 days and 7 days postoperatively.

The protein expression of pERK1/2 to tERK1/2 in different groups was determined by the ratio of bulk density (volume INT/mm^2^) at 4 and 7 days after intestinal mucosal I/R injuries. The values showed that pERK1/2 in MSCs group was significantly higher than that in the saline and sham groups at 4 days after intestinal I/R (both P<0.05, Figure **4**). This trend continued until 7days postoperatively (both P<0.05, Figure **4**). Results of western blot analyses showed that NF-kBp65 protein expression was at a lower level in sham group both at 4 and 7days postoperatively. However, protein expression of NF-kBp65 in saline group was significantly higher than that in MSCs group at 4 and 7days after intestinal I/R injury (P<0.05 and P<0.01 respectively, Figure **5**), which implied that much more inflammatory cytokines were activated in saline group.

## Discussion

Bone marrow derived mesenchymal stem cells (MSCs) are considered to be a potentially useful therapeutic option for treatment of a wide variety of ischemia/reperfusion disorders including intestinal I/R injury [27,28]. The mechanisms that mediate the effects of MSCs in ameliorating intestinal I/R injuries are however still unknown. The old dogma that administered MSCs engraft and differentiate into specialized cell types has been abandoned, and the proposition that therapeutic efficacy of MSCs is mediated via secretion of a variety of trophic cytokines and chemokines that stimulate regeneration and differentiation of injured cells [29], and inhibit proliferation of immune cells via secretion of anti-inflammatory cytokines [30] has gained popularity. In this study we first investigated the migrating characteristics of engrafted male MSCs after submucosal infusion in female recipient by Y chromosome *in situ* hybridization. We then speculated that the therapeutic effect of the homing MSCs against a complex I/R injuries was likely to be derived from the synergy of inhibiting inflammation and promoting regeneration by the administered MSCs, and that this efficacy was correlated with the amount of the engrafted cells. To assess the potential role of MSCs transplantation after intestinal I/R injuries, we focused on expression of proinflammatory cytokine TNF-α within intestinal mucosa and signaling pathways NF-kappaB after superior mesenteric artery I/R and evaluated the expression of PCNA and ERK1/2 in intestinal mucosa to clarify the therapeutic potential of infused MSCs.

Intestinal I/R injury was associated with prompt release of endotoxin into the portal venous circulation, followed by release of a significant amount of TNF-α into intestinal mucosa and systemic circulation [31]. TNF-α is a peptide mainly derived from stimulated macrophages and is implicated in the pathogenesis of multiple organ system and cell impairment associated with sepsis. Released TNF-α is a key proinflammatory cytokine which induce increased superoxide anion generation, leukotriene, increased neutrophil aggregation and adherence to endothelial cells, all relevant to cell and tissue injury [32]. In our experiment, mucosal TNF-α content in sham group was consistently at lower level throughout the observed time points postoperatively. However, intestinal mucosal TNF-α in both MSCs and saline groups increased to maximum values at day 1 postoperatively and there was no significant differences bwteen the two groups. With increasing postoperative time points, significant differences were noted between the group receiving MSCs and one receiving saline injection. Intestinal mucosal TNF-α in saline group was considerably higher than that in the MSCs group, both at 4 and 7days postoperatively (both P<0.01). Mucosal TNF-α levels in these two groups eventually dropped to approximate levels in the sham group at 10 days postoperatively, although the MSCs group had a more rapid drop compared to the saline group, and mucosal TNF-α content decreased to the approximate level of sham group at 7 days postoperatively (P>0.05, shown in Figure 2). These results indicated that the increasing magnitude of mucosal TNF-α was significantly inhibited by transplantation of MSCs and reduced TNF-α release lead to a lower inflammatory response hence less impairment to intestinal mucosal cells.

PCNA is a significant cell cycle-regulated nuclear protein for DNA-polymerase δ in eukaryotic cells [33]. Since DNA-polymerase δ is involved in resynthesis of excised damaged DNA strands during DNA repair. PCNA labeled nuclei identify cells in late G1 and early S phase of the cell cycle and its expression is increased during DNA repair and DNA synthesis process [34]. As a result, increasing expression of PCNA was an important symbol for both DNA synthesis and DNA repair after cellular damage. A previous study demonstrated that with increased expression of PCNA within intestinal mucosa, the repair process of I/R injured mucosal cells was significantly accelerated [35]. Our current findings suggest that at 4 and 7days after intestinal I/R impairment, expression of PCNA in MSCs group was significantly higher than that in saline and sham groups. This demonstrates that MSCs transplantation was a potential determinant of increased expression of mucosal PCNA which in turn accelerated restoration of damaged mucosal cells as well as replacement of dead absorptive epithelial cells. These results were consistent with those of a previous study by Munoz et al [36]. In that study, MSCs were topically administered into I/R injured mouse hippocampus and expression of PCNA was found to be considerably higher in hippocampus specimens of recipient mouse than the control group 4 days after MSCs infusion. In addition, hippocampus cell proliferation and neurological function was markedly improved in experimental group. These two studies underscore MSCs’ potential to promote cellular proliferation by accelerating the expression of PCNA after ischemia/reperfusion injury.

Intestinal mucosal epithelium continuously and rapidly regenerates itself throughout life. This rapid and dynamic renewal is maintained by crypt proliferating unit containing stem cells which give rise to four epithelial lineage cells [37]. The proliferation and differentiation processes of intestinal mucosal cells are accompanied by activation of a variety of cell signal pathways, and ERK1/2 is an important signaling pathway for mucosal cell proliferation [38,39]. Tissue renewal and cell regeneration are crucial for intestinal mucosal recovery after I/R injuries. El-Assal et al [40] revealed that activation of ERK1/2 resulted in accelerating intestinal mucosal cell hyperplasia and repair after I/R damage. Results of our study showed that the expression of phosphorylation ERK1/2 (pERK1/2) was at a relatively low level in the sham group postoperatively, which indicated that lesser injury factors resulted in lesser cellular proliferation. However, mucosal expression of pERK1/2 in saline and MSCs groups was clearly up-regulated after intestinal I/R injury due to the requirement of mucosal epithelial restoration, and this process was markedly enhanced by MSCs administration. Data shown in Figure 4 demonstrate that expression of pERK1/2 in MSCs group was significantly higher than that in the saline group at 4 and 7days postoperatively (both P<0.05) which indicate that MSCs may promote I/R injured intestinal mucosal restoration by up-regulating the expression of phosphorylation ERK1/2.

Recent evidence has proved that transcription factor nuclear factor-kappaB (NF-κB) plays a crucial role in regulating the expression of several genes involved in inflammatory response process, and some of these genes are activated during intestinal ischemia/reperfusion injury [41]. Activation of phosphorylation NF-κB may lead to enhanced expression of a series of proinflammatory genes that inevitably result in further cellular and tissue impairment. Previous studies have showed that inhibiting activation of NF-κB may attenuate tissue and cellular inflammatory response injuries [42,43]. Strategy for considering inhibition of NF-κB activation as a promising molecular target for ameliorating intestinal I/R injury has been deemed as a new therapeutic modality to improve prognosis after intestinal I/R injury. In this study, phosphorylation NF-κB activation was significantly inhibited by submucosal infusion of MSCs after superior mesenteric artery ischemia/reperfusion injury and the experimental results showed that mucosal NF-κB expression in sham group was at markedly lower level compared to the other two groups both at 4 and 7days postoperatively. At 4 days after intestinal I/R injury, mucosal NF-κB expression in saline group rapidly rose to a significantly high level in comparison with the sham group, but was significantly reduced in MSCs group (P<0.05). This trend became particularly obvious at 7 days after MSCs transplantation (P<0.01). These data demonstrate that MSCs have the potential of suppressing overexpression of NF-κB within intestinal mucosa after I/R injuries which in turn lead to lesser proinflammatory cytokines release and lesser mucosal impairment.

In summary, we investigated the anti-inflammatory and proliferative role of MSCs in an ischemia and reperfusion injured intestinal model and found that MSCs not only have the capacity to inhibit release of proinflammatory cytokines and suppress overexpression of proinflammatory genes, but also have the potential of accelerating expression of proliferative genes involved in intestinal mucosal cellular regeneration. Our study also demonstrated that MSCs alleviating intestinal ischemia/reperfusion injury was probably due to its capacity to reduce inflammatory cytokine release, inhibit proinflammatory gene activation and improve proliferation of injured mucosal cells. Further research is however needed to fully understand mechanisms by which MSCs exerts its influence on intestinal mucosal inflammatory and proliferative signal pathway.

## References

[1] Friedenstein AJ, Chailakhjan RK, Lalykina KS (1970) The development of fibroblast colonies in monolayer cultures of guinea-pig bonemarrow and spleen cells. Cell Tissue Kinet 3: 393-403. PubMed: 5523063. 5523063 10.1111/j.1365-2184.1970.tb00347.x

[2] Bianco P, Robey PG, Simmons PJ (2008) Mesenchymal stem cells: revisiting history, concepts, and assays. Cell Stem Cell 2: 313-319. doi:10.1016/j.stem.2008.03.002. PubMed: 18397751. 18397751 10.1016/j.stem.2008.03.002 PMC2613570

[3] Chamberlain G, Fox J, Ashton B, Middleton J (2007) Concise review: mesenchymal stem cells: their phenotype, differentiation capacity, immunological features, and potential for homing. Stem Cells 25: 2739-2749. doi:10.1634/stemcells.2007-0197. PubMed: 17656645. 17656645 10.1634/stemcells.2007-0197

[4] Pittenger MF, Mackay AM, Beck SC, Jaiswal RK, Douglas R et al. (1999) Multilineage potential of adult human mesenchymal stem cells. Science (80-) 284: 143-147 10.1126/science.284.5411.143 10102814

[5] Lee KD, Kuo TK, Whang-Peng J, Chung YF, Lin CT et al. (2004) In vitro hepatic differentiation of human mesenchymal stem cells. Hepatology 40: 1275-1284. doi:10.1002/hep.20469. PubMed: 15562440. 15562440 10.1002/hep.20469

[6] Taléns-Visconti R, Bonora A, Jover R, Mirabet V, Carbonell F et al. (2006) Hepatogenic differentiation of human mesenchymal stem cells from adipose tissue in comparison with bone marrow mesenchymal stem cells. World J Gastroenterol 12: 5834-5845. PubMed: 17007050. 17007050 10.3748/wjg.v12.i36.5834 PMC4100665

[7] Hisanaga E, Park KY, Yamada S, Hashimoto H, Takeuchi T et al. (2008) A simple method to induce differentiation of murine bone marrow mesenchymal cells to insulin-producing cells using conophylline and betacellulin-delta4. Endocr J 55: 535-543. doi:10.1507/endocrj.K07E-173. PubMed: 18480554. 18480554 10.1507/endocrj.k07e-173

[8] Chao KC, Chao KF, Fu YS, Liu SH (2008) Islet-like clusters derived from mesenchymal stem cells in Wharton’s Jelly of the human umbilical cord for transplantation to control type 1 diabetes. PLOS ONE 3: e1451. doi:10.1371/journal.pone.0001451. PubMed: 18197261. 18197261 10.1371/journal.pone.0001451 PMC2180192

[9] Zanini C, Bruno S, Mandili G, Baci D, Cerutti F et al. (2011) Differentiation of mesenchymal stem cells derived from pancreatic islets and bone marrow into islet-like cell phenotype. PLOS ONE 6: e28175. doi:10.1371/journal.pone.0028175. PubMed: 22194812. 22194812 10.1371/journal.pone.0028175 PMC3241623

[10] Lu P, Blesch A, Tuszynski MH (2004) Induction of bone marrow stromal cells to neurons: differentiation, transdifferentiation, or artifact. J Neurosci Res 77: 174-191. doi:10.1002/jnr.20148. PubMed: 15211585. 15211585 10.1002/jnr.20148

[11] Greco SJ, Zhou C, Ye JH, Rameshwar P (2007) An interdisciplinary approach and characterization of neuronal cells transdifferentiated from human mesenchymal stem cells. Stem Cells Dev 16: 811-826. doi:10.1089/scd.2007.0011. PubMed: 17999602. 17999602 10.1089/scd.2007.0011

[12] Nauta AJ, Fibbe WE (2007) Immunomodulatory properties of mesenchymal stromal cells. Blood 110: 3499-3506. doi:10.1182/blood-2007-02-069716. PubMed: 17664353. 17664353 10.1182/blood-2007-02-069716

[13] Zhao S, Wehner R, Bornhäuser M, Wassmuth R, Bachmann M et al. (2010) Immunomodulatory properties of mesenchymal stromal cells and their therapeutic consequences for immune-mediated disorders. Stem Cells Dev 19: 607-614. doi:10.1089/scd.2009.0345. PubMed: 19824807. 19824807 10.1089/scd.2009.0345

[14] Uccelli A, Laroni A, Freedman MS (2011) Mesenchymal stem cells for the treatment of multiple sclerosis and other neurological diseases. Lancet Neurol 10: 649-656. doi:10.1016/S1474-4422(11)70121-1. PubMed: 21683930. 21683930 10.1016/S1474-4422(11)70121-1

[15] Morigi M, Introna M, Imberti B, Corna D, Abbate M et al. (2008) Human bone marrow mesenchymal stem cells accelerate recovery of acute renal injury and prolong survival in mice. Stem Cells 26: 2075-2082. doi:10.1634/stemcells.2007-0795. PubMed: 18499895. 18499895 10.1634/stemcells.2007-0795

[16] Gonzalez-Rey E, Anderson P, González MA, Rico L, Büscher D et al. (2009) Human adult stem cells derived from adipose tissue protect against experimental colitis and sepsis. Gut 58: 929-939. doi:10.1136/gut.2008.168534. PubMed: 19136511. 19136511 10.1136/gut.2008.168534

[17] González MA, Gonzalez-Rey E, Rico L, Büscher D, Delgado M (2009) Adipose-derived mesenchymal stem cells alleviate experimental colitis by inhibiting inflammatory and autoimmune responses. Gastroenterology 136: 978-989. doi:10.1053/j.gastro.2008.11.041. PubMed: 19135996. 19135996 10.1053/j.gastro.2008.11.041

[18] Fisher-Shoval Y, Barhum Y, Sadan O, Yust-Katz S, Ben-Zur T et al. (2012) Transplantation of placenta-derived mesenchymal stem cells in the EAE mouse model of MS. J Mol Neurosci 48: 176-184. doi:10.1007/s12031-012-9805-6. PubMed: 22638856. 22638856 10.1007/s12031-012-9805-6

[19] Le Blanc K, Frassoni F, Ball L, Locatelli F, Roelofs H et al. (2008) Mesenchymal stem cells for treatment of steroid-resistant, severe, acute graft-versus-host disease: a phase II study. Lancet 371: 1579-1586. doi:10.1016/S0140-6736(08)60690-X. PubMed: 18468541. 18468541 10.1016/S0140-6736(08)60690-X

[20] Hare JM, Traverse JH, Henry TD, Dib N, Strumpf RK et al. (2009) A randomized, double-blind, placebo-controlled, dose-escalation study of intravenous adult human mesenchymal stem cells (prochymal) after acute myocardial infarction. J Am Coll Cardiol 54: 2277-2286. doi:10.1016/j.jacc.2009.06.055. PubMed: 19958962. 19958962 10.1016/j.jacc.2009.06.055 PMC3580848

[21] Duijvestein M, Vos AC, Roelofs H, Wildenberg ME, Wendrich BB et al. (2010) Autologous bone marrow-derived mesenchymal stromal cell treatment for refractory luminal Crohn’s disease: results of a phase I study. Gut 59: 1662-1669. doi:10.1136/gut.2010.215152. PubMed: 20921206. 20921206 10.1136/gut.2010.215152

[22] Collard CD, Gelman S (2001) Pathophysiology, clinical manifestations, and prevention of ischemia-reperfusion injury. Anesthesiology 94: 1133-1138. doi:10.1097/00000542-200106000-00030. PubMed: 11465607. 11465607 10.1097/00000542-200106000-00030

[23] Koike K, Moore FA, Moore EE, Read RA, Carl VS et al. (1993) Gut ischemia mediates lung injury by a xanthine oxidase-dependent neutrophil mechanism. J Surg Res 54: 469-473. doi:10.1006/jsre.1993.1072. PubMed: 8395621. 8395621 10.1006/jsre.1993.1072

[24] Jiang H, Qu L, Li Y, Gu L, Shi Y et al. (2011) Bone marrow mesenchymal stem cells reduce intestinal ischemia/reperfusion injuries in rats. J Surg Res 168: 127-134. doi:10.1016/j.jss.2009.07.035. PubMed: 19932900. 19932900 10.1016/j.jss.2009.07.035

[25] Pittenger MF, Mackay AM, Beck SC, Jaiswal RK, Douglas R et al. (1999) Multilineage potential of adult human mesenchymal stem cells. Science (80-) 284: 143-147 10.1126/science.284.5411.143 10102814

[26] Souza AL Jr, Poggetti RS, Fontes B, Birolini D (2000) Gut ischemia/reperfusion activates lung macrophages for tumor necrosis factor and hydrogen peroxide production. J Trauma 49: 232-236. doi:10.1097/00005373-200008000-00008. PubMed: 10963533. 10963533 10.1097/00005373-200008000-00008

[27] Giordano A, Galderisi U, Marino IR (2007) From the laboratory bench to the patient’s bedside: an update on clinical trials with mesenchymal stem cells. J Cell Physiol 211: 27-35. doi:10.1002/jcp.20959. PubMed: 17226788. 17226788 10.1002/jcp.20959

[28] Lovell MJ, Yasin M, Lee KL, Cheung KK, Shintani Y et al. (2010) Bone marrow mononuclear cells reduce myocardial reperfusion injury by activating the PI3K/Akt survival pathway. Atherosclerosis 213: 67-76. doi:10.1016/j.atherosclerosis.2010.07.045. PubMed: 20810112. 20810112 10.1016/j.atherosclerosis.2010.07.045

[29] Lee JW, Fang X, Krasnodembskaya A, Howard JP, Matthay MA (2011) Concise review: Mesenchymal stem cells for acute lung injury: role of paracrine soluble factors. Stem Cells 29: 913-919. doi:10.1002/stem.643. PubMed: 21506195. 21506195 10.1002/stem.643 PMC3293251

[30] Di Nicola M, Carlo-Stella C, Magni M, Milanesi M, Longoni PD et al. (2002) Human bone marrow stromal cells suppress T-lymphocyte proliferation induced by cellular or nonspecific mitogenic stimuli. Blood 99: 3838-3843. doi:10.1182/blood.V99.10.3838. PubMed: 11986244. 11986244 10.1182/blood.v99.10.3838

[31] Caty MG, Guice KS, Oldham KT, Remick DG, Kunkel SI (1990) Evidence for tumor necrosis factor-induced pulmonary microvascular injury after intestinal ischemia-reperfusion injury. Ann Surg 212: 694-700. doi:10.1097/00000658-199012000-00007. PubMed: 2175168. 2175168 10.1097/00000658-199012000-00007 PMC1358254

[32] Campolo M, Di Paola R, Impellizzeri D, Crupi R, Morittu VM et al. (2013) Effects of a polyphenol present in olive oil, oleuropein aglycone, in a murine model of intestinal ischemia/reperfusion injury. J Leukoc Biol 93: 277-287. doi:10.1189/jlb.0712317. PubMed: 23233730. 23233730 10.1189/jlb.0712317

[33] Prelich G, Tan CK, Kostura M, Mathews MB, So AG et al. (1987) Functional identity of proliferating cell nuclear antigen and a DNA polymerase-delta auxiliary protein. Nature 326: 517-520. doi:10.1038/326517a0. PubMed: 2882424. 2882424 10.1038/326517a0

[34] Prelich G, Stillman B (1988) Coordinated leading and lagging strand synthesis during SV40 DNA replication in vitro requires PCNA. Cell 53: 117-126. doi:10.1016/0092-8674(88)90493-X. PubMed: 2894900. 2894900 10.1016/0092-8674(88)90493-x

[35] Itoh H, Yagi M, Fushida S, Tani T, Hashimoto T et al. (2000) Activation of immediate early gene, c-fos, and c-jun in the rat small intestine after ischemia/reperfusion. Transplantation 69: 598-604. doi:10.1097/00007890-200002270-00022. PubMed: 10708117. 10708117 10.1097/00007890-200002270-00022

[36] Munoz JR, Stoutenger BR, Robinson AP, Spees JL, Prockop DJ (2005) Human stem/progenitor cells from bone marrow promote neurogenesis of endogenous neural stem cells in the hippocampus of mice. Proc Natl Acad Sci U S A 102: 18171-18176. doi:10.1073/pnas.0508945102. PubMed: 16330757. 16330757 10.1073/pnas.0508945102 PMC1312406

[37] Karam SM (1999) Lineage commitment and maturation of epithelial cells in the gut. Front Biosci 4: D286-D298. doi:10.2741/Karam. PubMed: 10077541. 10077541 10.2741/karam

[38] Ikeda M, Takei T, Mills I, Sumpio BE (1998) Calcium-independent activation of extracellular signal-regulated kinases 1 and 2 by cyclic strain. Biochem Biophys Res Commun 247: 462-465. doi:10.1006/bbrc.1998.8811. PubMed: 9642151. 9642151 10.1006/bbrc.1998.8811

[39] Li W, Duzgun A, Sumpio BE, Basson MD (2001) Integrin and FAK-mediated MAPK activation is required for cyclic strain mitogenic effects in Caco-2 cells. Am J Physiol Gastrointest Liver Physiol 280: G75-G87. PubMed: 11123200. 11123200 10.1152/ajpgi.2001.280.1.G75

[40] El-Assal ON, Besner GE (2005) HB-EGF enhances restitution after intestinal ischemia/reperfusion via PI3K/Akt and MEK/ERK1/2 activation. Gastroenterology 129: 609-625. doi:10.1053/j.gastro.2005.05.054. PubMed: 16083716. 16083716 10.1016/j.gastro.2005.05.054

[41] Nichols TC (2004) NF-kappaB and reperfusion injury. Drug News Perspect 17: 99-104. doi:10.1358/dnp.2004.17.2.829042. PubMed: 15098063. 15098063 10.1358/dnp.2004.17.2.829042

[42] Wu F, Chakravarti S (2007) Differential expression of inflammatory and fibrogenic genes and their regulation by NF-kappaB inhibition in a mouse model of chronic colitis. J Immunol 179: 6988-7000. PubMed: 17982090. 17982090 10.4049/jimmunol.179.10.6988

[43] Cheon JH, Kim JS, Kim JM, Kim N, Jung HC et al. (2006) Plant sterol guggulsterone inhibits nuclear factor-kappaB signaling in intestinal epithelial cells by blocking IkappaB kinase and ameliorates acute murine colitis. Inflamm Bowel Dis 12: 1152-1161. doi:10.1097/01.mib.0000235830.94057.c6. PubMed: 17119390. 17119390 10.1097/01.mib.0000235830.94057.c6

